# Monitoring of newborns at high risk for brain injury

**DOI:** 10.1186/s13052-016-0261-8

**Published:** 2016-05-14

**Authors:** Francesco Pisani, Carlotta Spagnoli

**Affiliations:** Child Neuropsychiatry Unit, Neuroscience Department, University of Parma, Via Gramsci 14, 43126 Parma, Italy

**Keywords:** Newborn, Neonatal neurology, Monitoring, EEG, Neuroimaging, Evoked potentials, Neonatal seizures, Stroke, Hypoxic-ischaemic encephalopathy, Diagnosis

## Abstract

Due to the increasing number of surviving preterm newborns and to the recognition of therapeutic hypothermia as the current gold standard in newborns with hypoxic-ischaemic encephalopathy, there has been a growing interest in the implementation of brain monitoring tools in newborns at high risk for neurological disorders.

Among the most frequent neurological conditions and presentations in the neonatal period, neonatal seizures and neonatal status epilepticus, paroxysmal non-epileptic motor phenomena, hypoxic-ischaemic encephalopathy, white matter injury of prematurity and stroke require specific approaches to diagnosis. In this review we will describe the characteristics, aims, indications and limitations of routinely available diagnostic techniques such as conventional and amplitude-integrated EEG, evoked potentials, cranial ultrasound and brain MRI. We will conclude by briefly outlining potential future perspectives from research studies.

## Background

The increasing number of preterm births with the associated risk of long term neurological sequelae [1] and, at the same time, the availability of new therapeutic strategies [2] determine a growing need for cerebral function monitoring in newborns who are at high risk of brain injury.

In this review, we will describe methods, timing, indications and aims of brain monitoring in newborns at risk for brain injury with the conventional techniques of monitoring currently used in newborns with neonatal seizures, hypoxic-ischaemic encephalopathy (HIE), intraventricular haemorrhage (IVH), periventricular leukomalacia and stroke.

For the purporses of this article, first of all we identified topics which, in our own experience, constitute the main aspects of brain monitoring in neonatal neurology, we then identified the most relevant and widely available techniques in clinical practice and we selected papers dealing with arbitrarily selected clinical issues (neonatal seizures, hypoxic-ischemic encephalopathy, stroke, prematurity), chosen for their epidemiological relevance in clinical practice. A PubMed search using the following search-terms was made: neonatal seizures, neonatal EEG, EEG monitoring, cranial ultrasound scan and newborn, cranial ultrasound and preterm newborn, MRI and newborn, evoked potentials and newborns. It was also integrated with sources found in the reviewed papers or from book chapters. We decided not to define a time frame in publication years because for some monitoring techniques we were unable to find relevant recent studies (i.e. evoked potentials). Therefore our review does not represent a systematic or comprehensive review of the literature but rather a way to convey some views and a “state-of-the art” on selected topics.

## Brain monitoring tools

### EEG and amplitude-EEG

There are many different clinical situations in which electroencephalogram (EEG) would be indicated. First of all, as it is non-invasive, it should be considered together with neurological examination, in case of concerns about the neonate’s neurological status or the possibility of seizures [3], especially if specific risk factors are present [4–7]. Neonatal seizures have been classified into subtle, clonic, myoclonic and tonic, based on their clinical characteristics [8]. However, clinical definition of neonatal seizures alone is not adequate as some clinical events might rather represent brain-stem release phenomena [9].

When investigating suspicious clinical events, EEG is necessary to exclude or confirm a diagnosis of neonatal seizures [10].

Neonatal seizures should be differentiated from paroxysmal non-epileptic motor phenomena. These are defined as movements standing out from the normal motor repertoire of newborns, for which there is no association with EEG changes [10]. In some cases, consideration of clinical characteristics of the paroxysmal event suffices for the differential diagnosis, while in others polygraphic video-EEG is mandatory [10, 11]. Subtle motor phenomena (such as cycling, pedalling or boxing) are of controversial interpretation: they were considered as brain-stem release phenomena or of spinal origin by Mizrahi [9]. but classified by Volpe as subtle seizures [12], because occasionally associated with epileptiform ictal discharges on EEG.

The use of conventional polygraphic video-EEG allows description of a series of clinically-relevant neurophysiological characteristics of neonatal seizures (definition of seizure onset and spread, seizure duration, correlation with motor phenomena). The onset is typically focal, especially in full-term newborns, while in preterm newborns both regional [13] and focal [14] onset have been commonly reported. Description of seizure onset is important in clinical practice as the consistent presence of a single onset focus is highly suggestive of focal injury, even though diffuse pathology can also manifest as focal discharges [13]. Furthermore, documentation of a spread from one hemisphere to the contralateral one has been associated with worse outcome and development of epilepsy [15].

However, after confirmation of neonatal seizures, prolonged or continuous monitoring is required for correct quantification of “seizure burden” and for neonatal status epilepticus diagnosis [4], in an attempt to contrast its unfavourable prognostic consequences [16, 17].

The diagnosis and the definition of neonatal status epilepticus (NSE) is still controversial. It has been usually defined either as continuous seizures lasting for more than 30 min or seizures present for at least 50 % of the recording time, with no return to the baseline neurologic condition between episodes [18]. An association with severe brain damage has been demonstrated and NSE is prognostically more detrimental than recurrent seizures [19, 20].

EEG is mandatory to monitor efficacy of anticonvulsants and for detection of the uncoupling phenomenon [21]. Consequently, continuation of EEG monitoring for at least 24 h of seizure freedom has been recommended. With the advent of continuous EEG monitoring, it has become apparent that a great bulk of neonatal seizures are electrographic-only [22].

Furthermore, the assessment of background activity with conventional EEG allows documentation of brain injury and evaluation of its degree of severity, especially using serial recordings. This represents a strong predictor of outcome [4], independent of aetiology.

With the availability of therapeutic hypothermia, there has been a great impulse into the spreading of amplitude-integrated EEG (aEEG) use in neonatal intensive care units (NICU). Among its biggest strengths are the easiness of use by neonatologists [23], the compressed time scale and the reduced montage, which shorten the time required for application and interpretation of tracings.

Although reliability in assessing background activity and degree of HIE, with good correlation with outcome, has been demonstrated [24], aEEG has to be considered as a screening tool [4, 25] for seizure detection, and reference to conventional or “raw” EEG should be made in order to increase its sensitivity and specificity whenever suspicious neonatal seizures are detected [26, 27]. In conclusion, aEEG limitations must be taken into account to correctly use it: for example it might miss brief seizures or seizures originating far from recording electrodes (false negative results) or generate false positives due to artefacts [5, 25, 28].

### EEG in the evaluation of prematurity

In preterm newborns, EEG can be used to estimate the timing of a brain insult and its severity degree with serial recordings beginning soon after birth [29].

Acute stage abnormalities reflect the acute phase of brain injury. EEG is mainly characterised by changes in continuity, amplitude and dominating frequencies. On the contrary, two different patterns are recognised as chronic stage abnormalities: the dysmature and the disorganized pattern. The dysmature pattern is characterised by the presence of EEG features that would be physiological for an infant 2 weeks or more younger than the conceptional age (CA) of the patient [30, 31]. It is typically reported following prolonged mild acute stage abnormalities without ultrasonographic changes or with IVH without parenchymal involvement [31].

A disorganised pattern describes a background activity characterised by distorted delta waves associated with abnormal sharp waves [31]. It is mainly reported in infants between 32 and 36 weeks of CA [29] and in association with white matter injury, sometimes pre-dating cranial ultrasound evidence [32, 33]. It is associated with the subsequent development of cerebral palsy [29, 31].

The literature recommends to start with a first recording in the immediate post-natal period, in order to establish the timing of injury. Knowledge on both the severity of acute stage abnormalities and the type and severity of chronic stage abnormalities can improve prognostication. Tracings with increased discontinuity and decreased amplitude are associated with unfavourable prognosis [34] and show the best sensitivity in the first two days of life [35].

### EEG in the evaluation of HIE

An algorithm for the EEG surveillance of full-term newborns with HIE has been proposed [36]. Different time points have been considered as the best predictors of normal outcome in different studies: within the first 12 h of life [36], or within 24–36 h [37]. Time-appropriate evaluation of background activities is paramount for correct planning of subsequent recordings, and for optimal prognostic accuracy [38], as tracings tend to progressively improve with time [39]. In fact, one single normal EEG obtained more than one week after an hypoxic-ischemic event has no prognostic value [39]. On the contrary, a severely abnormal EEG can be related to an unfavourable outcome if recorded from 12 h of life, although a follow-up EEG after 24 h is helpful to rule out potential confounding factors [36]. Finally, in case of intermediate findings in the initial EEG, a control EEG during the first week of life is important for prognostic purposes [36].

The accurate assessment of the severity of background EEG abnormalities in the first 6 h of life has become one of the criteria to select newborns for therapeutic hypothermia [40], even if this task is usually accomplished by means of aEEG [3]. With the spreading in clinical use of therapeutic hypothermia, it has become clear that it determines a slower recovery rate of background EEG activity compared to normothermic condition [41]. While the time to recovery of a normal background activity on aEEG in the normothermic newborn is predictive of a favourable outcome within 24 h, in the hypothermic newborn 48 h are necessary. Similarly, while the reappearance of sleep-wake cycle within 36 h is a marker of good prognosis, in hypothermic newborns there is a shift to up to 60 h after birth [41].

### EEG in the evaluation of stroke

EEG can allow early detection of focal cerebral injury [42] in the context of perinatal stroke. EEG might point to a focal structural aetiology by identifying focal electrographic seizures, focal sharp waves or focal attenuation of background activities [5], which would prompt neuroimaging. A recent study on perinatal arterial ischemic stroke confirmed suppression over the infarcted side, together with the presence of unilateral theta bursts intermixed with sharp or spike waves. Seizure patterns characteristically consisted of focal sharp, spikes/polispikes at 1–2Hz and a predominance of electrographic-only seizures [43].

### Standard or Continuous conventional EEG?

Conventional polygraphic video-EEG represents the current gold standard for seizure diagnosis and quantification in newborns, as it allows evaluation of the site of onset and propagation of seizures [4] and provides detailed information on background activity, reflecting brain maturation and presence of ongoing or previous brain injury.

Conventional EEG can be performed as either a standard EEG or as a continuous or prolonged recording. Standard neonatal EEG recordings differ from the ones undertaken at older ages because a complete cycle of wakefulness, active and quiet sleep should be recorded in newborns aged ≥ 30 weeks CA, and the most continuous and discontinuous patterns in younger newborns should be obtained for at least 40 min [29, 44]. These recommendations are motivated by the higher sensitivity of quiet sleep for background abnormalities detection.

Continuous cEEG has now been advocated as the preferable monitoring tool in high risk newborns, especially to correctly quantify neonatal seizures. In this case, continuous monitoring has been recommended for at least the first 24 h of life, as the majority of seizures would be identified within this time frame [45–47], although some researchers have proven an increased risk in the first 24–36 h period [45–47] and additional studies reported seizures in preterm newborns to occur later than in full-term ones, secondary to the timing of IVH [20]. Furthermore, it was demonstrated within 48 h from birth in only approximately 10 % of preterm newborns below 29 weeks CA and in 50 % of those ≥30 weeks, while the mean onset time in the first group was 8.3 days versus 3.2 days in the second [48].

## Evoked potentials

Evoked potentials have been more extensively applied in neonatal HIE. In this context, somatosensory evoked potentials (SSEP) show a positive predictive value of 70–100 % for short-term outcome prediction [49, 50]. While absent or deteriorating visual evoked potentials (VEP) are associated with unfavourable outcomes [49].

The predictive value of normal SSEP seems to be superior to their absence in newborns [49], but they can represent a good indicator of unfavourable motor outcome if absent on a 4 weeks follow-up [51]. The role of the bilateral absence of cortical SSEP in predicting cerebral palsy has been confirmed in newborns with neonatal encephalopathy in both term and preterm newborns [51].

The combined application of VEP and SSEP seems to give the best prognostic accuracy [49], especially in case of moderate HIE.

Based on this knowledge, some authors suggested recording of VEP first. If abnormal, SSEP are unlikely to add clinically-relevant information, whereas if they are normal, SSEP are recommended in order to refine prognostic accuracy [49].

After the advent of therapeutical hypothermia, some authors suggest caution in interpreting SSEP as in older studies undertaken on normothermic newborns [52].

VEP can also be used in preterm newborns to predict death and motor deficits. One study demonstrated a sensitivity and specificity of flash VEP of 86 % at birth and of 88 % at term equivalent age for survival and of 60 and 92 % for the development of cerebral palsy [53].

Early brainstem auditory evoked potentials (BAEP) are of limited prognostic value, because of the great variability in latencies [54] and the high frequency of transient abnormalities in the acute phase [55].

## Neuroimaging

### Cranial ultrasound and MRI

Cranial ultrasonography is the most common brain imaging technique in NICUs, due to its easiness of use and the possibility to perform serial studies at a low cost [56]. It is especially sensitive in detecting IVH, ventriculomegaly and focal cystic periventricular leukomalacia (PVL), while more subtle lesions, such as those involving the white matter, might not be detected [57].

Performing a cranial ultrasound at the time of admission is paramount to gather evidence of lesions of prenatal onset or of congenital abnormalities, especially if there is no history of perinatal asphyxia. Afterwards, serial exams are advised in order to detect lesions soon after they occur, especially in the preterm infant, and then to monitor their evolution in order to diagnose complications such as ventriculomegaly and PVL. At present, cystic PVL is more commonly localised other than extensive, and these smaller cysts, usually appearing at 3–6 weeks after injury onset, usually resolve within several weeks and would not be appreciated on a brain magnetic resonance imaging (MRI) performed at term-equivalent age, when they are substituted by ventriculomegaly [58–61]. The possibility of an occurrence of a late onset PVL should also be borne in mind especially in case of acute deterioration, which should prompt the execution of additional ultrasound scans. Pre-discharge cranial ultrasound is also highly recommended [56].

Standard cranial ultrasound procedures are based on the use of the anterior fontanelle as the main acoustic window however, for specific situations, supplementary acoustic windows are required to reach adequate sensitivity. Using the posterior fontanel enables visualisation of the occipital horns of the lateral ventricles, of the occipital parenchyma, the tentorium and cerebellum. Specific indications have been suggested, especially for early detection of cerebellar haemorrhages, congenital malformations and visualisation of the 4th ventricle and cisterna magna [56]. Additionally, Doppler ultrasound techniques, spectral Doppler with resistive index (RI) calculation, colour and power Doppler techniques can be applied to evaluate intracranial vessels (patency, flow, cerebral vascular autoregulation, hyperhaemia) [62]. It can be used to diagnose cerebral sinovenous thrombosis (CSVT) if the superior sagittal sinus is occluded, but MRI and magnetic resonance (MR) venography are often necessary to confirm diagnosis [63].

However, cranial ultrasound examination has some limitations such as: poor visualisation of the brain convexity, of small abscesses and encephalitis, risk of missing small arterial cortical infarction and watershed lesions, underdiagnosis of hypoglycemic occipital parenchymal injury (unless the posterior fontanel is used), and underdetermined evaluation of the posterior limb of the internal capsule.

Therefore, brain MRI should be recommended in the above-mentioned clinical pictures and in the other clinical context as described in the following sections.

The most used MRI sequences in newborns have been summarised in a recent review [64].

## Prematurity

### Cranial ultrasound in the evaluation of prematurity

A scanning protocol has been proposed for preterm infants, with variations depending on gestational age [65]. From the 23rd to the 35th week of GA, a cranial ultrasound scan is always recommended on day one of life and at one week of life, the at 2 and 3 weeks. For newborns with a GA between 23 and 26 wGA, additional scanning should be undertaken on the 2nd and 3rd day of life, weekly until 312 wGA, then on alternate weeks until 36 wGA. For preterm newborns born between 27 and 29 wGA after scanning weekly up to 31 wCA, one additional follow-up cranial ultrasound should be undertaken at 36 wCA. For preterm neonates born ≥ 29 wGA, scanning at 1 and 3 weeks is recommended. Irrespective of GA, one last cranial ultrasound scan is advisable at term equivalent age. Almost all haemorrhages develop within the first week after birth [59]. After establishing this diagnosis, involvement of the white matter and/or the cerebellum should be looked for. Additionally, sequential scanning is recommended in order to promptly detect post-haemorrhagic ventricular dilatation. The ventricular index, the anterior horn width and the thalamo-occipital distance should be measured [66, 67].

### Brain MRI in the preterm newborn

In case of uncomplicated IVH in the preterm newborn, integration between cranial ultrasound scans and conventional brain MRI performed at term-equivalent age can be especially useful to evaluate the development of ventriculomegaly and to detect associated white matter injury and cerebellar involvement [57], which can consist of either haemorrhage or infarction, the latter of which is now considered as one of the preterm patterns of injury.

The best timing for brain scanning is represented by term equivalent age [64]. Prognostication and comparison of severity degree between patients and studies has been improved with the development of scoring systems for either white matter or grey matter injuries [68–71].

## Cranial ultrasound in the full-term newborn

The two main patterns of injury in the full-term newborn comprise: predominantly deep grey matter injury, in which areas of hyperechogenicity develop after 24–72 h from birth, first involving the thalami, and the watershed pattern, which is difficult to recognise due to the involvement of the convexity.

## Brain MRI in the full-term newborn

In HIE, the “acute near total asphyxia” pattern of injury is more easily demonstrated with diffusion weighted imaging (DWI) in the first week of life and only by the end of the first week with conventional imaging [72]. Similarly, the watershed predominant pattern of injury can be appreciated as a loss of grey-white matter differentiation on conventional MRI, but can be documented more easily and earlier with DWI. Brain MRI can also be very useful in central nervous system (CNS) infection, in some cases enabling recognition of specific patterns [63, 73, 74]. Additionally, brain MRI is the gold standard for recognition and classification of cerebral malformations, thanks to the high resolution multiplanar imaging it provides [75].

### Neuroimaging in the evaluation of stroke

Arterial infarction might be detected in the (near) term newborn by ultrasound scans as an increased or asymmetrical echogenicity between the two hemispheres, but brain MRI is usually required. Arterial ischemic infarction in a cortical branch of a major cerebral artery is initially documented as a loss of gray/white matter differentiation, mainly with a low signal intensity on T1 and a high signal intensity on T2 weighted images. Diffusion weighted images are particularly helpful in stroke identification in the first week, while between the second and sixth week there is, at the beginning, an increase in white/matter differentiation followed by widening of the extracerebral spaces and formation of a porencephalic cyst. After the 6th week, the affected hemisphere is usually smaller and shows a decrease in myelination [76].

## Perspectives from research studies

In the neurophysiologic field, additional monitoring strategies have been applied in the neonatal period but still need further studies. These include digital trend analysis (DSA), envelope trend and spectrogram [77]. Density spectral array shows spectral power of specific electrodes or an averaged power from a group of electrodes. It can be used in addition to c-EEG, although its diagnostic contribution has to be elucidated [4]. Experience with envelope trend (displaying the median amplitude of given EEG epochs) [4] is even more limited [77], even though a sensitivity approaching that of aEEG in case of prolonged seizures has been reported in one study [78].

Automatic detection systems have also been developed, for background grading [79, 80] and for neonatal seizure recognition, in this last case either based on video [81–83] or on EEG analysis [84]. These still represent research tools, as only a few centres have used them in the clinical setting [25]. Finally, video-processing based techniques are also under evaluation for apnoeas simulation [85] and estimation of respiratory rate [86].

Another field of research particularly applied in preterm newborns is represented by near infrared spectroscopy (NIRS), used to study haemodynamics and cerebral oxygenation [87].

Other research groups are also working to evaluate the role of high density EEG in improving information acquisition on the immature brain [88].

Advanced MRI techniques, already part of medical practice, such as diffusion tensor imaging (DTI) and spectroscopy can provide more quantitative information than conventional imaging [89], increasing prognostic accuracy or widening the spectrum of clinical questions that can be answered from early-on.

## Conclusions

We reviewed neurophysiologic and neuroimaging tools to be used in evaluating newborns at high risk of neurological sequelae. We reported on the best timing, indication and aims of their use according to the available scientific literature. We recommend integration of EEG, ultrasound, MRI and, when available, evoked potentials data in order to achieve accurate prognostication.

## Abbreviations

aEEG: amplitude-integreted EEG
BAEP: brainstem auditory evoked potentials
CA: conceptional age
cEEG: conventional EEG
CNS: central nervous system
CSVT: cerebral sinovenous thrombosis
DSA: digital trend analysis
DTI: diffusion tensor imaging
DWI: diffusion weighted imaging
EEG: electroencephalogram
HIE: hypoxic-ischaemic encephalopathy
IVH: intraventricular haemorrhage
MR: magnetic resonance
MRI: magnetic resonance imaging
NICU: neonatal intensive care units
NIRS: near infrared spectroscopy
NSE: neonatal status epilepticus
PVL: periventricular leukomalacia
RI: resistence index
SSEP: somatosensory evoked potentials
VEP: visual evoked potential

## References

[1] Johnson S, Evans TA, Draper ES, Field DJ, Manktelow BN, Marlow N, Matthews R, Petrou S, Seaton SE, Smith LK, Boyle EM. Neurodevelopmental outcomes following late and moderate prematurity: a population-based cohort study. Arch Dis Child Fetal Neonatal Ed. 2015;100(4):F301–8. 10.1136/archdischild-2014-307684 PMC4484499 25834170

[2] Jacobs SE, Berg M, Hunt R, Tarnow-Mordi WO, Inder TE, Davis PG (2013). Cooling for newborns with hypoxic ischaemic encephalopathy. Cochrane Database Syst Rev.

[3] Cilio MR (2009). EEG and the newborn. J Pediatric Neurology.

[4] Shelhaas RA, Chang T, Tsuchida T, Scher MS, Riviello JJ, Abend NS (2011). The American clinical neurophysiology society's guideline on continuous electroencephalography monitoring in neonates. J Clin Neurophysiol.

[5] McCoy B, Hahn CD (2013). Continuous EEG monitoring in the neonatal intensive care unit. J Clin Neurophysiol.

[6] Shah DK, Zempel J, Barton T, Lukas K, Inder TE (2010). Electrographic seizures in preterm infants during the first week of life are associated with cerebral injury. Pediatr Res.

[7] Gupta SN, Kechli AM, Kanamalla US (2009). Intracranial hemorrhage in term newborns: management and outcomes. Pediatr Neurol.

[8] Lombroso CT (1996). Neonatal seizures: a clinician's overview. Brain Dev.

[9] Mizrahi EM, Kellaway P (1987). Characterization and classification of neonatal seizures. Neurology.

[10] Orivoli S, Facini C, Pisani F (2015). Paroxysmal nonepileptic motor phenomena in newborn. Brain Dev.

[11] Hahn JS, Sanger T (2004). Neonatal movement disorders. Neoreviews.

[12] Clancy RR, Legido A (1987). The exact ictal and interictal duration of electroencephalographic neonatal seizures. Epilepsia.

[13] Patrizi S, Holmes GL, Orzalesi M, Allemand F (2003). Neonatal seizures: characteristics of EEG ictal activity in preterm and fullterm infants. Brain Dev.

[14] Okumura A, Hayakawa F, Kato T, Itomi K, Maruyama K, Kubota T (2008). Ictal electroencephalographic findings of neonatal seizures in preterm infants. Brain Dev.

[15] Pisani F, Copioli C, Di Gioia C, Turco E, Sisti L (2008). Neonatal seizures: relation of ictal video-electroencephalography (EEG) findings with neurodevelopmental outcome. J Child Neurol.

[16] Lawrence R, Inder T (2010). Neonatal status epilepticus. Semin Pediatr Neurol.

[17] Pavlidis E, Spagnoli C, Pelosi A, Mazzotta S, Pisani F (2015). Neonatal status epilepticus: differences between preterm and term newborns. Eur J Paediatr Neurol.

[18] Wusthoff CJ (2013). Diagnosing neonatal seizures and status epilepticus. J Clin Neurophysiol.

[19] Pisani F, Cerminara C, Fusco C, Sisti L (2007). Neonatal status epilepticus vs recurrent neonatal seizures: clinical findings and outcome. Neurology.

[20] Pisani F, Piccolo B, Cantalupo G, Copioli C, Fusco C, Pelosi A (2012). Neonatal seizures and postneonatal epilepsy: a 7-y follow-up study. Pediatr Res.

[21] Scher MS, Alvin J, Gaus L, Minnigh B, Painter MJ (2003). Uncoupling of EEG-clinical neonatal seizures after antiepileptic drug use. Pediatr Neurol.

[22] Murray DM, Boylan GB, Ali I, Ryan CA, Murphy BP, Connolly S (2008). Defining the gap between electrographic seizure burden, clinical expression and staff recognition of neonatal seizures. Arch Dis Child Fetal Neonatal Ed.

[23] Boylan G, Burgoyne L, Moore C, O'Flaherty B, Rennie J (2010). An international survey of EEG use in the neonatal intensive care unit. Acta Paediatr.

[24] Lamblin MD, Walls Esquivel E, André M (2013). The electroencephalogram of the full-term newborn: review of normal features and hypoxic-ischemic encephalopathy patterns. Neurophysiol Clin.

[25] Abend NS, Wusthoff CJ (2012). Neonatal seizures and status epilepticus. J Clin Neurophysiol.

[26] Shah DK, Mackay MT, Lavery S, Watson S, Harvey AS, Zempel J (2008). Accuracy of bedside electroencephalographic monitoring in comparison with simultaneous continuous conventional electroencephalography for seizure detection in term infants. Pediatrics.

[27] Zimbric MR, Sharpe CM, Albright KC, Nespeca MP (2011). Three-channel electroencephalogram montage in neonatal seizure detection and quantification. Pediatr Neurol.

[28] Shah DK, Boylan GB, Rennie JM (2012). Monitoring of seizures in the newborn. Arch Dis Child Fetal Neonatal Ed.

[29] Watanabe K, Hayakawa F, Okumura A (1999). Neonatal EEG: a powerful tool in the assessment of brain damage in preterm infants. Brain Dev.

[30] Ngueyn The Tich S, d’Allest AM, Villepin AT, de Belliscize J, Walls-Esquivel E, Salefranque F (2007). Pathological features of neonatal EEG in preterm babies born before 30 weeks of gestationnal age. Neurophysiol Clin.

[31] Okumura A, Hayakawa F, Kato T, Kuno K, Watanabe K (2002). Developmental outcome and types of chronic-stage EEG abnormalities in preterm infants. Dev Med Child Neurol.

[32] Tsuji T, Okumura A, Kidokoro H, Hayakawa F, Kubota T, Maruyama K, et al. Differences between periventricular hemorrhagic infarction and periventricular leukomalacia. Brain Dev. 2014;36:555–62. 10.1016/j.braindev.2013.07.014 23978489

[33] Castro Conde JR, Martínez ED, Campo CG, Pérez AM, McLean ML (2004). Positive temporal sharp waves in preterm infants with and without brain ultrasound lesions. Clin Neurophysiol.

[34] Kidokoro H, Okumura A, Hayakawa F, Kato T, Maruyama K, Kubota T, Suzuki M, Natsume J, Watanabe K, Kojima S. Chronologic changes in neonatal EEG findings in periventricular leukomalacia. Pediatrics. 2009;124(3):e468–75. 10.1542/peds.2008-2967 19706584

[35] Maruyama K, Okumura A, Hayakawa F, Kato T, Kuno K, Watanabe K (2002). Prognostic value of EEG depression in preterm infants for later development of cerebral palsy. Neuropediatrics.

[36] Lamblin MD, André M (2011). Electroencephalogram of the full-term newborn. Normal features and hypoxic-ischemic encephalopathy. Neurophysiol Clin.

[37] Murray DM, Boylan GB, Ryan CA, Connolly S (2009). Early EEG findings in hypoxic-ischemic encephalopathy predict outcomes at 2 years. Pediatrics.

[38] Tsuchida TN, Wusthoff CJ, Shellhaas RA, Abend NS, Hahn CD, Sullivan JE (2013). American clinical neurophysiology society standardized EEG terminology and categorization for the description of continuous EEG monitoring in neonates: report of the American Clinical Neurophysiology Society critical care monitoring committee. J Clin Neurophysiol.

[39] Tsuchida TN (2013). EEG background patterns and prognostication of neonatal encephalopathy in the era of hypothermia. J Clin Neurophysiol.

[40] Papile LA, Baley JE, Benitz W, Cummings J, Carlo WA, Committee on Fetus and Newborn (2014). Hypothermia and neonatal encephalopathy. Pediatrics.

[41] Thoresen M, Hellström-Westas L, Liu X, de Vries LS (2010). Effect of hypothermia on amplitude-integrated electroencephalogram in infants with asphyxia. Pediatrics.

[42] Chang T, Tsuchida TN (2014). Conventional (continuous) EEG monitoring in the NICU. Curr Pediatr Rev.

[43] Low E, Mathieson SR, Stevenson NJ, Livingstone V, Ryan CA, Bogue CO (2014). Early postnatal EEG features of perinatal arterial ischaemic stroke with seizures. PLoS One.

[44] Holmes GL, Moshé SL, Royden Jones H (2006). Clinical neurophysiology of infancy, childhood, and adolescence.

[45] Naim MY, Gaynor JW, Chen J, Nicolson SC, Fuller S, Spray TL, Dlugos DJ, Clancy RR, Costa LV, Licht DJ, Xiao R, Meldrum H, Abend NS. Subclinical seizures identified by postoperative electroencephalographic monitoring are common after neonatal cardiac surgery. J Thorac Cardiovasc Surg. 2015;150(1):169–78. 10.1016/j.jtcvs.2015.03.045 PMC4936910 25957454

[46] Laroia N, Guillet R, Burchfiel J, McBride MC (1998). EEG background as predictor of electrographic seizures in high-risk neonates. Epilepsia.

[47] Nash KB, Bonifacio SL, Glass HC, Sullivan JE, Barkovich AJ, Ferriero DM (2011). Video-EEG monitoring in newborns with hypoxic-ischemic encephalopathy. Neurology.

[48] Pisani F, Barilli AL, Sisti L, Bevilacqua G, Seri S (2008). Preterm infants with video-EEG confirmed seizures: outcome at 30 months of age. Brain Dev.

[49] Taylor MJ, Murphy WJ, Whyte HE (1992). Prognostic reliability of somatosensory and visual evoked potentials of asphyxiated term infants. Dev Med Child Neurol.

[50] Scalais E, François-Adant A, Nuttin C, Bachy A, Guérit JM (1998). Multimodality evoked potentials as a prognostic tool in term asphyxiated newborns. Electroencephalogr Clin Neurophysiol.

[51] Suppiej A, Cappellari A, Franzoi M, Traverso A, Ermani M, Zanardo V (2010). Bilateral loss of cortical somatosensory evoked potential at birth predicts cerebral palsy in term and near-term newborns. Early Hum Dev.

[52] Garfinkle J, Sant'Anna GM, Rosenblatt B, Majnemer A, Wintermark P, Shevell MI (2015). Somatosensory evoked potentials in neonates with hypoxic-ischemic encephalopathy treated with hypothermia. Eur J Paediatr Neurol.

[53] Shepherd AJ, Saunders KJ, McCulloch DL, Dutton GN (1999). Prognostic value of flash visual evoked potentials in preterm infants. Dev Med Child Neurol.

[54] Hyde ML, Matsumoto N, Alberti PW (1987). The normative basis for click and frequency-specific BERA in high-risk infants. Acta Otolaryngol.

[55] Stockard JE, Stockard JJ, Kleinberg F, Westmoreland BF (1983). Prognostic value of brainstem auditory evoked potentials in neonates. Arch Neurol.

[56] van Wezel-Meijler G, Steggerda SJ, Leijser LM (2010). Cranial ultrasonography in neonates: role and limitations. Semin Perinatol.

[57] de Vries LS, Benders MJ, Groenendaal F (2013). Imaging the premature brain: ultrasound or MRI?. Neuroradiology.

[58] de Vries LS, van Haastert IL, Rademaker KJ, Koopman C, Groenendaal F. Ultrasound abnormalities preceding cerebral palsy in high-risk preterm infants. J Pediatr 2004;144:815-20. 10.1016/j.jpeds.2004.03.034 15192633

[59] Leijser LM, de Bruine FT, Steggerda SJ, van der Grond J, Walther FJ, van Wezel-Meijler G (2009). Brain imaging findings in very preterm infants throughout the neonatal period: part I. Incidences and evolution of lesions, comparison between ultrasound and MRI. Early Hum Dev.

[60] Groenendaal F, Termote JUM, Heide-Jalving M, van Haastert IC, de Vries LS (2010). Complications affecting preterm neonates from 1991 to 2006: what have we gained?. Acta Paediatr.

[61] Sarkar S, Shankaran S, Laptook AR, Sood BG, Do B, Stoll BJ, Van Meurs KP, Bell EF, Das A, Barks J; Generic Database Subcommittee of the Eunice Kennedy Shriver National Institute of Child Health and Human Development Neonatal Research Network. Screening cranial imaging at multiple time points improves cystic periventricular leukomalacia detection. Am J Perinatol. 2015;32(10):973–9. 10.1055/s-0035-1545666 PMC4697863 25730135

[62] Daneman A, Epelman M, Blaser S, Jarrin JR (2006). Imaging of the brain in full-term neonates: does sonography still play a role?. Pediatr Radiol.

[63] Weeke LC, Groenendaal F, Toet MC, Benders MJ, Nievelstein RA, van Rooij LG, de Vries LS. The aetiology of neonatal seizures and the diagnostic contribution of neonatal cerebral magnetic resonance imaging. Dev Med Child Neurol. 2015;57:248–56. 10.1111/dmcn.12629 25385195

[64] Kwon SH, Vasung L, Ment LR, Huppi PS (2014). The role of neuroimaging in predicting neurodevelopmental outcomes of preterm neonates. Clin Perinatol.

[65] Leijser LM, de Vries LS, Cowan FM (2006). Using cerebral ultrasound effectively in the newborn infant. Early Hum Dev.

[66] Brouwer MJ, de Vries LS, Groenendaal F, Koopman C, Pistorius LR, Mulder EJ, Benders MJ. New reference values for the neonatal cerebral ventricles. Radiology. 2012;262(1):224–33. 10.1148/radiol.11110334 22084208

[67] Brouwer MJ, de Vries LS, Pistorius L, Rademaker KJ, Groenendaal F, Benders MJ (2010). Ultrasound measurements of the lateral ventricles in neonates: why, how and when? A systematic review. Acta Paediatr.

[68] Chau V, Synnes A, Grunau RE, Poskitt KJ, Brant R, Miller SP (2013). Abnormal brain maturation in preterm neonates associated with adverse developmental outcomes. Neurology.

[69] Anderson PJ, Cheong JL, Thompson DK (2015). The predictive validity of neonatal MRI for neurodevelopmental outcome in very preterm children. Semin Perinatol.

[70] Holland D, Chang L, Ernst TM, Curran M, Buchthal SD, Alicata D, Skranes J, Johansen H, Hernandez A, Yamakawa R, Kuperman JM, Dale AM. Structural growth trajectories and rates of change in the first 3 months of infant brain development. JAMA Neurol. 2014;71(10):1266–74. 10.1001/jamaneurol.2014.1638 PMC4940157 25111045

[71] Kidokoro H, Anderson PJ, Doyle LW, Woodward LJ, Neil JJ, Inder TE (2014). Brain injury and altered brain growth in preterm infants: predictors and prognosis. Pediatrics.

[72] Bednarek N, Mathur A, Inder T, Wilkinson J, Neil J, Shimony J (2012). Impact of therapeutic hypothermia on MRI diffusion changes in neonatal encephalopathy. Neurology.

[73] de Vries LS, Verboon-Maciolek MA, Cowan FM, Groenendaal F (2006). The role of cranial ultrasound and magnetic resonance imaging in the diagnosis of infections of the central nervous system. Early Hum Dev.

[74] Lequin MH, Vermeulen JR, van Elburg RM, Barkhof F, Kornelisse RF, Swarte R, Govaert PP. Bacillus cereus meningoencephalitis in preterm infants: neuroimaging characteristics. AJNR Am J Neuroradiol. 2005;26:2137–43. PMC8148855 16155172

[75] Tortori-Donati P, Rossi A. Congenital malformations in the neonate. In: Rutherford MA, editor. MRI of the neonatal brain. http://www.mrineonatalbrain.com/. Last Accessed 22 Nov 2015.

[76] Mercuri E, Dubowitz L, Rutherford MA. Cerebral infarction in the full-term infant. In: Rutherford MA,editor. MRI of the neonatal brain. http://www.mrineonatalbrain.com/. Last Accessed 22 Nov 2015.

[77] Riviello JJ (2013). Digital trend analysis in the pediatric and neonatal intensive care units. J Clin Neurophysiol.

[78] Abend NS, Dlugos D, Herman S (2008). Neonatal seizure detection using multichannel display of envelope trend. Epilepsia.

[79] Ahmed R, Temko A, Marnane W, Lightbody G, Boylan G (2015). Grading hypoxic-ischemic encephalopathy severity in neonatal EEG using GMM supervectors and the support vector machine. Clin Neurophysiol.

[80] Murphy K, Stevenson NJ, Goulding RM, Lloyd RO, Korotchikova I, Livingstone V, Boylan GB. Automated analysis of multi-channel EEG in preterm infants. Clin Neurophysiol. 2015;126:1692–702. 10.1016/j.clinph.2014.11.024 25538005

[81] Karayiannis NB, Tao G, Frost JD, Wise MS, Hrachovy RA, Mizrahi EM (2006). Automated detection of videotaped neonatal seizures based on motion segmentation methods. Clin Neurophysiol.

[82] Pisani F, Spagnoli C, Pavlidis E, Facini C, Kouamou Ntonfo GM, Ferrari G, Raheli R. Real-time automated detection of clonic seizures in newborns. Clin Neurophysiol. 2014;125:1533–40. 10.1016/j.clinph.2013.12.119 24602566

[83] Ntonfo GM, Ferrari G, Raheli R, Pisani F (2012). Low-complexity image processing for real-time detection of neonatal clonic seizures. IEEE Trans Inf Technol Biomed.

[84] Mathieson SR, Stevenson NJ, Low E, Marnane WP, Rennie JM, Temko A, Lightbody G, Boylan GB. Validation of an automated seizure detection algorithm for term neonates. Clin Neurophysiol. 2015. doi:10.1016/j.clinph.2015.04.075. 10.1016/j.clinph.2015.04.075 PMC4727504 26055336

[85] Alinovi D, Cattani L, Ferrari G, Pisani F, Raheli R. Video simulation of apnoea episodes. IEEE International Conference on Multimedia Expo Workshops (ICMEW). 2015.

[86] Alinovi D, Cattani L, Ferrari G, Pisani F, Raheli R (2015). Spatio-temporal video processing for respiratory rate estimation.

[87] Toet MC, Lemmers PM (2009). Brain monitoring in neonates. Early Hum Dev.

[88] Roche-Labarbe N, Aarabi A, Kongolo G, Gondry-Jouet C, Dümpelmann M, Grebe R (2008). High-resolution electroencephalography and source localization in neonates. Hum Brain Mapp.

[89] Rennie JM, Kendall GS (2015). Advanced magnetic resonance techniques provide more accurate prognostic information than conventional imaging. Dev Med Child Neurol.

